# Ageism and older people’s health and well-being during the Covid-19-pandemic: the moderating role of subjective aging

**DOI:** 10.1007/s10433-021-00624-8

**Published:** 2021-04-30

**Authors:** Anna E. Kornadt, Isabelle Albert, Martine Hoffmann, Elke Murdock, Josepha Nell

**Affiliations:** 1 Department of Behavioural and Cognitive Sciences, University of Luxembourg, 11, Porte des Sciences, 4366 Esch-sur-Alzette, Luxembourg; 2 RBS–Center fir Altersfroen, 5955 Itzig, Luxembourg

**Keywords:** Ageism, Subjective aging, Covid-19, Well-being, Subjective health, Life satisfaction

## Abstract

In the Covid-19 pandemic, being older means being in a special focus: Probabilities for severe infections and mortality rise with increasing age and protective measures for this population group have been increased. This was accompanied by public discourse that portrayed older adults stereotypically as vulnerable and frail but also highlighted the hardships younger people have to endure to protect them. Given the possibly detrimental effects of ageism on individuals and societies, we were interested in older adults’ perception of ageism in the Corona-crisis and its relation to their health and well-being. Furthermore, we were interested in subjective aging variables as moderators in the ageism–health relationship. In June 2020, *N* = 611 independently living people aged 60 + from the Grand Duchy of Luxembourg were recruited via a survey research institute and interviewed online or by phone. They reported on perceived ageism in different contexts, their life satisfaction, subjective health, subjective age and self-perceptions of aging. Depending on context, ageism was perceived by around 20% of participants, and overall negatively related to subjective health and life satisfaction after the onset of the pandemic. Moderated hierarchical regressions showed that a younger subjective age buffered the negative effect of ageism on subjective health, while perceiving aging as social loss increased its effect on life satisfaction. We discuss the importance of addressing and reducing ageism (not only) in times of crisis and the consequences for individuals and societies.

## Introduction

The Covid-19 pandemic proves to be challenging for people and societies in various forms. One demographic group that had to deal with particular hardships were older people. Right from the beginning of the pandemic, it was communicated that people over the age of 60 had a higher risk of severe progression of the disease and also increased rates of mortality if contracting the virus (e.g., Onder et al. 2020). This group-based risk assessment (which, for instance, neglects the role of underlying health conditions, e.g. Montero-Odasso et al. 2020) had pronounced consequences for those included in the risk group: They were particularly advised to strictly follow protocols of protective measures, such as sheltering-in-place and physical distancing (including ceasing physical contact with children and grandchildren), with all the accompanying consequences for their daily lives and well-being (e.g., Krendl and Perry 2020; van Tilburg et al. 2020).

As a positive response to this situation, intergenerational solidarity and support in neighborhoods and institutions emerged, offering help with chores, for instance (e.g., Monahan et al. 2020). On the other hand, public discourse turned towards a narrative of grouping all people over the age of 60 together, into a group requiring shielding and mostly well-meant advice was offered on how this group should behave in the crisis. At the same time, advice was given to other demographic groups on how they should behave in order to protect older people, and—related to this—defend scarce medical and economic resources. This was accompanied by public display of pity and patronizing, but also anger directed towards older people. Taken together, these instances showcase pronounced ageism (Ayalon et al. 2020; Lichtenstein 2020; Morrow-Howell and Gonzalez 2020), culminating in reports that in some of the most strongly affected countries and regions, older people did not receive intensive care or ventilation therapy when resources were scarce and doctors had to perform triage decisions under pressure (Cesari and Proietti 2020).

Irrespective of such extreme examples, the perception and experience of ageism can potentially have negative consequences for older adults’ health and well-being (e.g., Chang et al. 2020; Monahan et al. 2020), a relationship that might even be exacerbated in times of crisis. Thus, we were interested in the perception of ageism by people over the age of 60 and how it was related to their self-rated health and life satisfaction during the first wave of the Covid-crisis in Luxembourg. Since the consequences of perceived ageism might differ according to people’s subjective aging experiences and perceptions of aging, a second question of our study was in how far self-perceptions of aging and subjective age moderated the ageism–outcome relationship.

### Ageism and its consequences for individuals and societies

Ageism, which can be defined as “*stereotypes, prejudice, or discrimination against (but also in favor of) people because of their chronological age*” (Ayalon and Tesch-Römer 2017, p. 1) can take various forms, and be displayed at different levels, within individuals, organizations, and cultures. At the heart of it is that people are perceived and treated in a certain way because they (are perceived to) belong to a certain age group, irrespective of their actual personal and individual characteristics. While ageism can also take benevolent forms (e.g., Chasteen et al. 2017), it is mostly rooted in the fact that older age is seen as a state of deterioration, loss of functioning and even senescence (Ayalon and Tesch-Römer 2018). Studies show that societally prevalent ageism comes with massive costs for health-care systems and economies (e.g. for the US, Levy et al. 2020). At the individual level, Chang and colleagues (2020) demonstrated in a recent meta-analysis including 422 studies from 45 countries, that ageism transported by institutions, cultural practices, individual perceptions and behaviors negatively impacted all investigated health outcomes (e.g. mortality, hospitalization, quality of life, health behaviors).

Importantly, not only objective instances of ageism are consequential, but the mere perception of being disadvantaged and treated unfairly due to ones’ age can also be detrimental (Bratt et al. 2018; Vauclair et al. 2015; Vogt Juan 2007). Being confronted with people’s negative perceptions of age might not only directly decrease developmental opportunities of older people, but also turn into self-fulfilling prophecies by becoming entrenched in people’s self, bodies and behaviors as they age (Levy 2009; Kornadt et al. 2020). So irrespective of the pandemic context, perceived ageism represents an important risk factor for health and well-being in later life. In a study with data from the European Social Survey, Vauclair and colleagues (2015) showed that perceived ageism predicted self-rated health above and beyond other variables, such as social capital (cf. Armeta et al. 2017). Relatedly, people’s life satisfaction is affected by perceived ageism (Garstka et al. 2004). Given that self-rated health and life satisfaction are frequently used and easily assessable variables with high importance for development in later life, we use both indicators as outcome variables in the present study.

### Manifestations of ageism during the pandemic

Only 10 months into the pandemic as of the time of writing, there has already been an abundance of papers in scientific journals and other outlets detailing the Covid-related increase of ageism. In one of the first review articles on the topic, Ayalon and colleagues (2020) impressively detailed instances of ageism that were found in newspapers all over the world. Those ranged from naming the virus “Boomer Remover” to suggesting that older adults should sacrifice themselves for the younger generations and the economy (see also examples in Meisner 2020; Wahl and Ehni 2020). Messages disseminated followed two trajectories: The “vulnerability narrative” focused on the group of older people as a uniform, undifferentiated group of fragile, vulnerable people, who should be protected at any cost. The “burden narrative”, on the other hand, focused on the hardships that younger people had to endure because they had to protect older people (Cohn-Schwartz and Ayalon 2020). More anecdotal evidence reported in the reviews and appeals was supported by systematic analyses. In an analysis of newspapers and other media outlets in Australia, the UK and the US, Liechtenstein (2020) showed that in all three countries, ageist and age-centric debates were prevalent, especially at the onset of the pandemic. (Xiang et al. 2020) investigated more than 80.000 tweets with a machine learning approach and found that on average 20% of these tweets had ageist content, devaluing older adults’ lives or ridiculing them. It has to be noted, however, that the most extreme of these posts (e.g. the suggestions by a Texan politician that older adults should sacrifice their lives if it will mitigate the pandemic’s economic damage to younger people) were also met with broad opposition and critique (Barrett et al. 2020).

Yet besides those narratives and articles describing the phenomenon, few papers have empirically dealt with the perception of ageism by older people themselves and the consequences as well as moderators of this relationship. Cohn-Schwartz and Ayalon (2020) for example asked Israeli adults aged 50+ whether they perceive ageism to be prevalent in Israeli society during the crisis, and found this was the case especially for the idea that older people are particularly vulnerable. About one quarter of their participants also reported that they experienced ageism in the health care system during the pandemic. In another study from Israel, Bergman et al. (2020) found that participants with higher self-reported ageism had higher anxiety levels during the pandemic. However, both studies did not directly look at the relationship of people’s own experiences of perceived ageism with health outcomes and what might moderate this relationship.

### Perceptions of subjective aging as moderators of the ageism–health relationship

Besides ageism directed at people by others or towards others, perceptions of subjective aging play a central role for personal development, health and well-being across the lifespan (Kornadt et al. 2020). Subjective aging is defined as “*individuals’ experiences with their own aging process and the state of being old*” and it includes self-perceptions of aging, as well as subjective age (Wurm et al. 2017, p. 28). Whereas self-perceptions of aging include ideas about one’s own aging process, subjective age is defined as the age one feels like relative to one’s chronological age (Kotter-Gruhn et al. 2016). Both variables have been shown to impact health, well-being and mortality of older people in a large number of studies with a variety of different operationalizations, samples and outcomes (for a meta-analysis see Westerhof et al. 2014). Their relevance in the Covid-19 pandemic was also demonstrated in one of the first empirical studies on well-being during the crisis. Losada-Baltar and colleagues (2020) found in their large sample of Spaniards aged 18–88 that people with more negative perceptions of their own aging (as measured with the widely used Atitudes Toward Own Aging scale by Lawton), perceived higher loneliness and more psychological distress in the first weeks of lockdown.

Besides the direct effect of subjective aging on health outcomes, those perceptions can also function as moderator in the relationship between predictors and health outcomes in later life. In the context of post-traumatic stress disease (PTSD) for example, people who felt comparatively older had a stronger negative relationship between PTSD symptoms and for instance physical functioning (Shrira et al. 2018). Feeling older also increased the relationship between depressive symptoms and physical morbidity in a longitudinal study (Segel-Karpas et al. 2017). Relatedly, self-perceptions of aging as physical decline moderated the use of self-regulatory strategies in case of a serious health event, with those participants that perceived aging more as physical decline engaging in less strategies (Wurm et al. 2013). (Han 2018) also found that declining functional health led to more depressive symptoms if people had more negative self-perceptions of aging. With regard to the relationship between ageism, health and well-being, the assumption that self-perceptions of aging and subjective age might act as moderators has actually been demonstrated in experimental studies (e.g., Weiss et al. 2013): For those people who feel they belong to the older age group (as indicated by higher subjective age) and also focus on the negative characteristics of aging (as indicated by higher perceptions of aging as physical decline and social loss), experiences of ageism might possess more self-relevance and threat potential and thus also affect them more strongly (Levy 2009).

It has to be noted that views on aging are a domain-specific construct (for an overview, see Kornadt et al. 2020). Besides, views on aging show stronger relationships to outcomes that match their respective content, this has been termed the stereotype matching effect (Levy and Leifheit-Limson 2009). Domain-specificity thus needs to be taken into account when linking different views on aging to various outcome variables.

### Aims and hypotheses of the current study

**Fig. 1:**
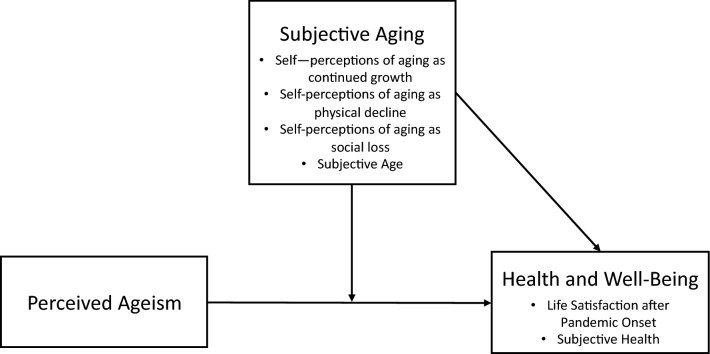
Schematic depiction of investigated relationships between perceived ageism and health and well-being outcomes, moderated by subjective aging variables

Given the notable increase and prevalence of ageism during the Covid-19 pandemic and the detrimental consequences that ageism might have on the health and well-being of older adults themselves, but also on a society, we were interested in the perception of ageism during the first wave of the Covid-crisis in Luxembourg. We asked people aged 60 and older if they had been treated unfairly due to their age in different contexts during the pandemic. We expected higher perceptions of ageist treatment to be related to worse subjective health and life satisfaction (H1). Furthermore, we were interested in the moderating role of self-perceptions of aging in different domains as well as subjective age for the relationship between ageism and outcomes. We expected a younger subjective age and self-perceptions of aging as continued growth to have a positive effect on subjective health and life satisfaction (H2a), but also a buffering effect in the relationship between ageism and life satisfaction (H3a). In contrast, an older subjective age and self-perceptions of aging as social loss and physical decline should be negatively related to subjective health and life satisfaction (H2b), and also exacerbate the relationship between ageism and the outcome variables (H3b). According to theories of stereotype matching (Levy and Leifheit-Limson 2009), we expected a more pronounced relationship between self-perceptions of aging as physical decline and subjective health, and self-perceptions of aging as social loss and life satisfaction (H4). Investigated relationships between all variables are schematically presented in Fig. 1.

## Method

### Sample and procedure

Our sample consisted of *N* = 611 community-dwelling participants aged 60–98 (*M*_age_ = 69.92, *SD* = 6.97). 49.6% of the sample were female and 29.5% reported at least some tertiary education. They were recruited in June 2020 by a survey research institute (TNS ILRES) in Luxembourg. The survey was carried out either by phone, for which participants were recruited via random digit dialing (*n* = 240, response rate 27%), or online, recruited from a large database of persons who agreed to be contacted for online surveys (*n* = 371, response rate 40%). Participants who completed the survey on the phone were older [t(606) = 6.94, *p *< 0.001], reported less perceived ageism [t(607) = − 4.08, *p* < 0.001], lower subjective health t(607) = − 3.25, *p* = 0.001], and lower life satisfaction before Corona [t(606) = − 2.04, *p* = 0.04]. There were no significant differences for the subjective aging variables. The questionnaire assessed sociodemographic information, the perception of the Covid-crisis in Luxembourg in general, their personal situation in the crisis, perceived ageism, subjective aging, and a number of other risk and resilience factors. The study was approved by the Ethics Review Panel of the University of Luxembourg (ERP 20-042-C CRISIS).

### Measures

#### Perceived ageism

We assessed perceived ageism by asking people “During the Covid-19 pandemic, have you felt that you were treated unfairly due to your age in the following domains”: 1) media coverage, 2) health care, 3) activities of daily life (e.g. shopping), and 4) within my social network (friends, family).^1^ These contexts were selected because of their relevance in previous research on ageism (e.g., Chasteen et al. 2017) and due to their relevance in the context of the pandemic. Participants had to indicate whether they felt unfairly treated very strongly, strongly, somewhat or not at all. We collapsed these items into one index of perceived ageism (Cronbach’s α = 0.75) and recoded the items so that higher values represent more perceived ageism.

#### Self-perceptions of aging

Self-perceptions of aging were assessed with the established AgeCog scales (Steverink et al. 2001) which assess people’s perception of their own aging (“Aging means to me…”) in the domains of physical decline (3 items, e.g.”…that my health is declining”, α = 0.80), continued growth (three items, e.g., “…that I continue to make plans”, α = 0.72) and social loss (four items, e.g. “…that I feel lonely more often”, α = 0.67). Participants had to rate the items on a four-point scale from completely applies to does not apply at all. Values were also recoded so that higher values represent higher endorsement of the respective scale.

#### Subjective age

Subjective age was assessed with one item asking people “Aside from your actual age: How old do you feel, in years?”. This number was subtracted from people’s chronological age, with more negative values indicating feeling younger than one’s chronological age. According to conventions, values of three standard deviations above and below the mean were removed (people who felt more than 38 years younger or more than 18 years older than their chronological age, 1.3% of cases).

#### Subjective health

Participants had to indicate their current state of health on a five-point scale ranging from very good to very bad. Again, values were recoded so that higher values indicated better subjective health.

#### Life satisfaction before and since the onset of the pandemic

Participants were asked to rate how satisfied they were with their life as a whole 1) before the spread of the Corona-Virus, and 2) since the spread of the Corona-Virus, respectively, on a four-point scale from very satisfied to very unsatisfied. Values were recoded so that higher values indicate more satisfaction.

### Analyses

We first computed descriptive statistics and correlations for the variables of interest to address means and bivariate relationships. To address whether perceived ageism was related to subjective health and life satisfaction and whether self-perceptions of aging and subjective age moderated the relationship between ageism and the outcome variables, we ran hierarchical moderated regression analyses with IBM SPSS 26 for both outcome variables separately. In a first step, age (and in the case of life satisfaction, satisfaction before the onset of the pandemic) were regressed on subjective health and life satisfaction after the onset of the pandemic, respectively. In a second step, perceived ageism was added as a predictor, in a third step the four subjective aging variables, and in a fourth step the interaction terms between ageism and the subjective aging variables. All predictor variables were standardized prior to analyses and the computation of interaction terms. When interactions were significant, we repeated the analyses for this predictor alone with the process macro (Hayes 2017) in order to probe the interaction. Due to listwise deletion of missing values, analysis N for the moderated regressions was *N* = 527 for the analysis with life satisfaction as outcome and *N* = 528 for the analyses with subjective health as outcome.

## Results

### Descriptive statistics and bivariate relations

Means and standard deviations as well as bivariate correlations between all study variables are presented in Table 1. On a mean level, participants reported relatively little ageism (*M* = 1.27, *SD* = 0.50), mostly in the media (*M* = 1.35, *SD* = 0.76), medical treatment (*M* = 1.32, *SD* = 0.72), and activities of daily life (*M* = 1.26, *SD* = 0.63), least in their social networks (*M* = 1.15, *SD* = 0.49). The overall perception of ageism was unrelated to age (*r* = − 0.03, *p* = 0.55). In absolute numbers, however, a considerable number of people reported to have been at least somewhat treated unfairly due to their age during the pandemic (media: 20,7%; health care: 19%; daily activities: 17,5%; family: 10,8%).

**Table 1:** Correlations and descriptive statistics

Variable	*n*	*M [95% CI]*	*SD*	*MD*	1	2	3	4	5	6	7	8	9
1. Age	608	69.92 [69.36, 70.47]	6.97	69.00	–								
2. Discrimination index	609	1.27 [1.23, 1.31]	0.50	1.00	− 0.03	–							
3. SPA Social loss	611	1.73 [1.68, 1.78]	0.61	1.75	0.10*	0.27**	–						
4. SPA Continued growth	609	2.90 [2.85, 2.95]	0.66	3.00	− 0.23**	− 0.08*	− 0.29**	–					
5. SPA Physical decline	611	2.53 [2.47, 2.59]	0.74	2.67	0.13**	0.10*	0.39**	− 0.29**	–				
6. Subjective age	532	− 10.03 [− 10.68, − 9.38]	7.59	− 10.00	− 0.10*	0.05	0.12**	− 0.22**	0.26**	-			
7. LS before Corona	608	3.26 [3.22, 3.20]	0.50	3.00	− 0.06	− 0.08*	− 0.20**	0.19**	− 0.17**	− 0.07	–		
8. LS after Corona	605	3.05 [3.01, 3.10]	0.54	3.00	− 0.06	− 0.35**	− 0.35**	0.25**	− 0.25**	− 0.12**	0.54**	–	
9. Subjective health	609	4.03 [3.98, 4.09]	0.73	4.00	−0 .13**	− 0.14**	− 0.28**	0.27**	−0 .50**	−0 .26**	0.21**	0.34**	–

*LS* Life satisfaction, *SPA* Self-perceptions of aging

^*^p < .05. ^**^p < .01. ^***^p < .001

Participants reported to be satisfied with their life, however, compared to their life satisfaction before the onset of the pandemic (*M* = 3.26, *SD* = 0.50), participants reported less life satisfaction after the onset of the pandemic (*M* = 3.05, *SD* = 0.54), *F*(1, 604) = 105.70, *p* < 0.001, η_p_^2^ = 0.15. Subjective health was rated overall to be good (*M* = 4.03, *SD* = 0.73). With regard to subjective aging, participants felt around 10 years younger than their actual age, and perceived their own aging to be rather accompanied by continued growth and physical decline and only somewhat by social loss.

Higher perceived ageism was related to lower life satisfaction before and after the onset of the pandemic, and also to worse subjective health. In addition, more perceived ageism was related to perceiving aging as being accompanied by more social loss and physical decline but was unrelated to the perception of continued growth and participants’ subjective age. The subjective aging variables were also related to subjective health and life satisfaction, with more continued growth, and lower subjective age as well as less physical decline and social loss accompanied with better subjective health and life satisfaction before and after Corona. Subjective age, however, was unrelated to life satisfaction before Corona.

### Moderated hierarchical linear regressions

#### Life satisfaction

**Fig. 2:**
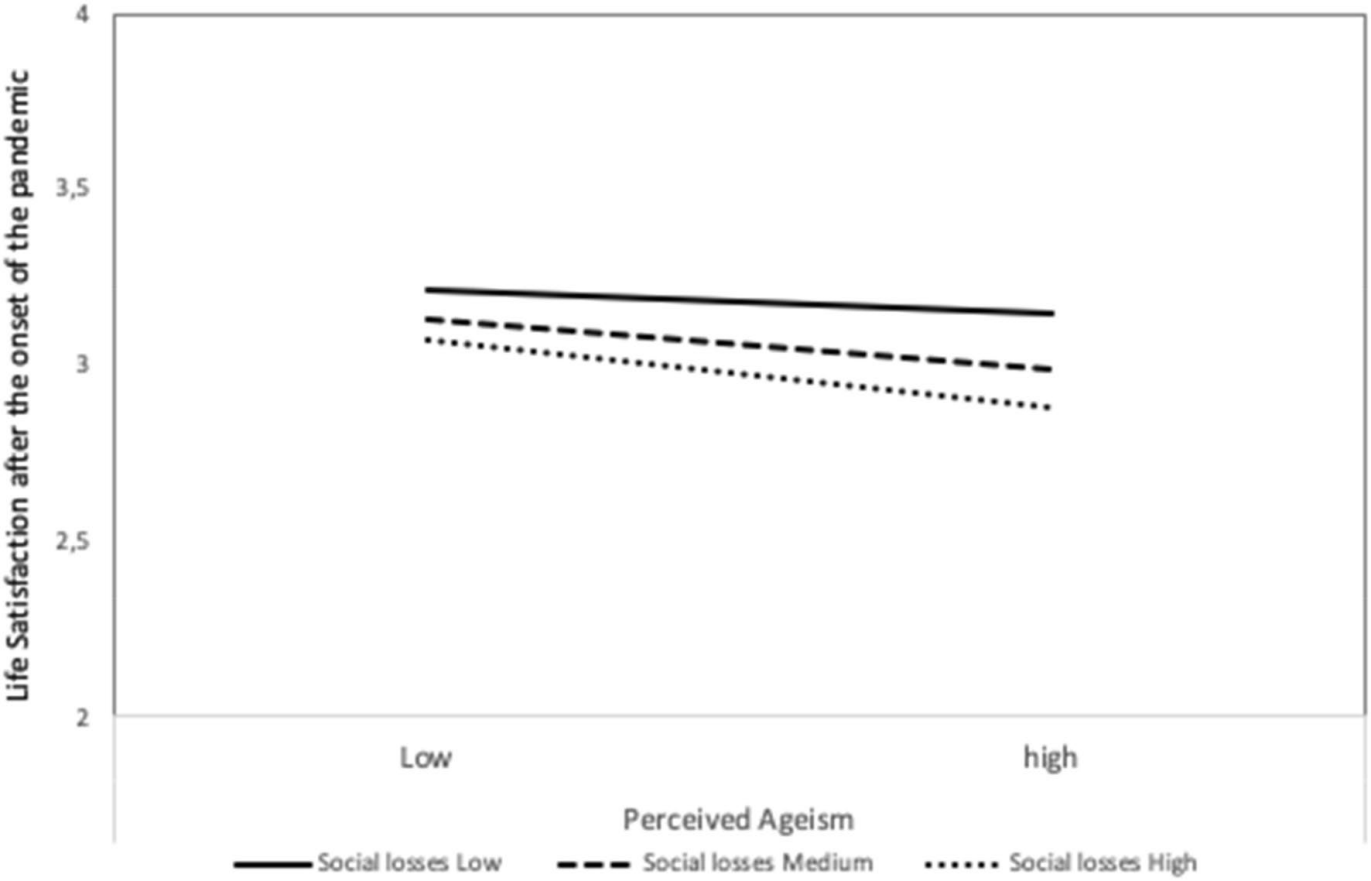
Effect of perceived ageism on life satisfaction after the onset of the pandemic for different values of self-perceptions of aging as social loss (low = 16th percentile, medium = 50th percentile, high 84th percentile)

Results of the analyses with life satisfaction after the onset of the Corona pandemic as outcome variable are displayed in Table 2. Controlling for age and life satisfaction before Corona,^2^ perceived ageism was still negatively related to life satisfaction after the onset of the pandemic (β = − 0.30, *p* < 0.001). Including the subjective aging variables significantly increased the amount of explained variance. In particular, perceiving one’s own aging as accompanied by more social loss was negatively related to life satisfaction (β = − 0.16, *p* < 0.001), whereas the perception of continued growth was positively related to life satisfaction (β = 0.10, *p* = 0.006). Entering the interactions between perceived ageism and the subjective aging variables yielded more explained variance and a significant interaction between social loss and ageism: For those who perceived their aging as accompanied by more social loss, the experience of ageism during the crisis had an especially negative effect on life satisfaction (Fig. 2).

**Table 2:** Hierarchical regression results with life satisfaction since the beginning of the crisis as outcome

Variable	*B*	*SE B*	β	*p*	*R*^*2*^	*p*	Δ*R*^*2*^
*Step 1*					0.27	< 0.001	0.27*
Constant	1.54	0.25		< 0.001			
LS before Corona	0.56	0.04	0.52	< 0.001			
Age	− 0.00	0.00	− 0.05	0.149			
*Step 2*					0.36	< 0.001	0.09*
Constant	1.63	0.23		< 0.001			
LS before Corona	0.54	0.04	0.50	< 0.001			
Age	− 0.01	0.00	− 0.06	0.083			
Discrimination index	− 0.17	0.02	− 0.30	< 0.001			
*Step 3*					0.41	< 0.001	0.05*
Constant	1.57	0.23		< 0.001			
LS before Corona	0.48	0.04	0.45	< 0.001			
Age	− 0.00	0.00	− 0.02	0.614			
Discrimination index	− 0.14	0.02	− 0.25	< 0.001			
SPA social loss	− 0.09	0.02	− 0.16	< 0.001			
SPA continued growth	0.06	0.02	0.10	0.006			
SPA physical decline	− 0.03	0.02	− 0.06	0.140			
Subjective age	− 0.01	0.02	− 0.01	0.712			
*Step 4*					0.42	0.015	0.01*
Constant	1.54	0.23		< 0.001			
LS before Corona	0.48	0.04	0.45	< 0.001			
Age	− 0.00	0.00	− 0.01	0.743			
Discrimination index	− 0.11	0.02	− 0.20	< 0.001			
SPA Social loss	− 0.09	0.02	− 0.16	< 0.001			
SPA Continued growth	0.06	0.02	0.10	0.007			
SPA Physical decline	− 0.03	0.02	− 0.05	0.187			
Subjective age	− 0.01	0.02	− 0.02	0.645			
Discr x Social loss	− 0.07	0.02	− 0.13	0.005			
Discr x Continued growth	0.01	0.02	0.00	0.667			
Discr x Physical decline	0.00	0.02	0.01	0.869			
Discr x Subjective age	− 0.01	0.02	− 0.02	0.474			

*LS* Life satisfaction, *SPA* Self-perceptions of aging; * *p* < .05

#### Subjective health

**Fig. 3:**
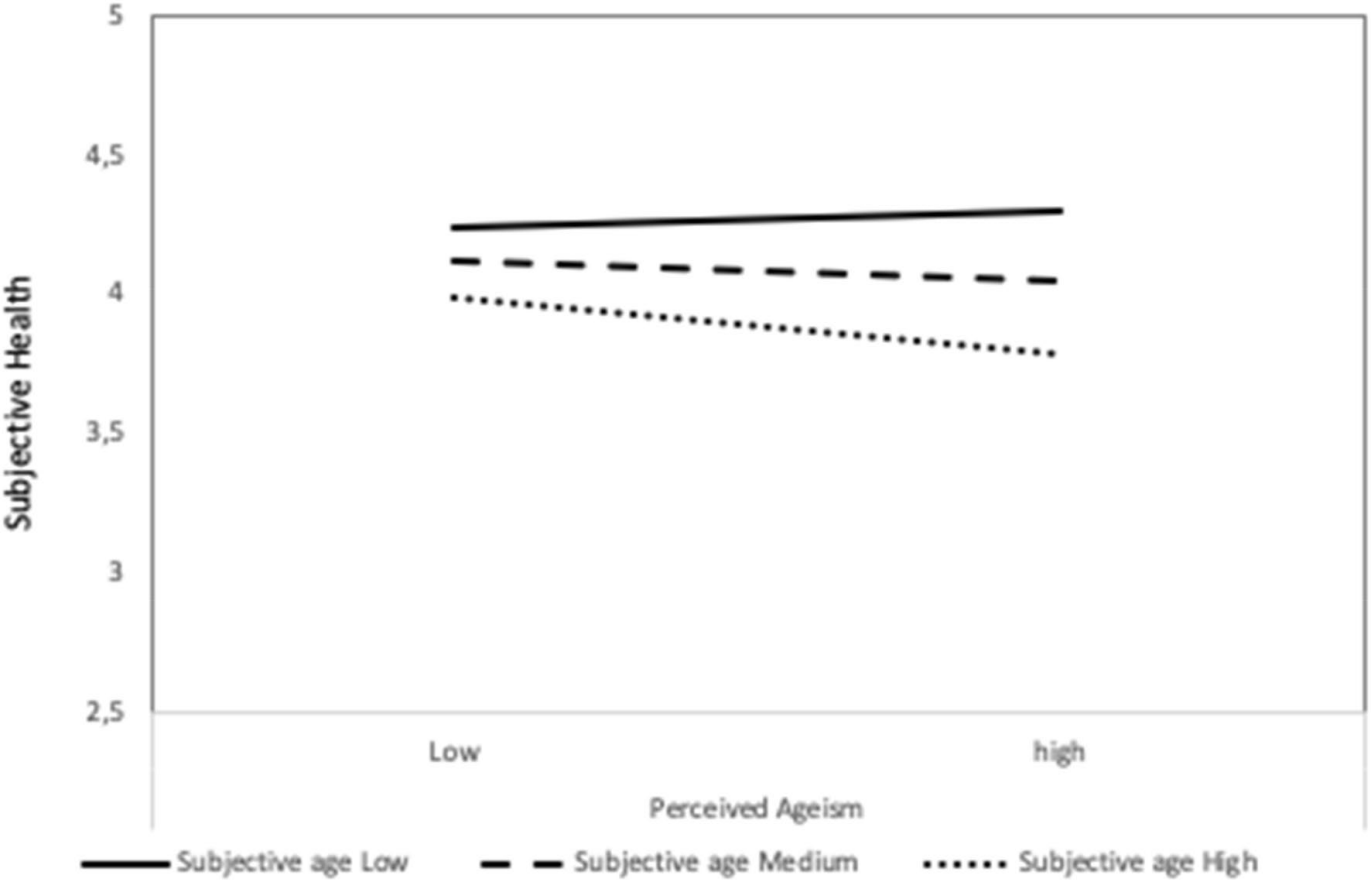
Effect of perceived ageism on subjective health for different values of subjective age (low = 16th percentile, medium = 50th percentile, high 84th percentile)

Results for subjective health as the outcome variable are displayed in Table 3. Controlling for age, perceived ageism was negatively related to subjective health (β = − 0.12, *p* = 0.004). The subjective aging variables incrementally explained variance in subjective health, perceiving aging accompanied by physical decline (β = − 0.40, *p* < 0.001) and an older subjective age (β = − 0.14, *p* < 0.001) were related to decreased subjective health. Again, including the interaction terms increased explained variance. Those who perceived more ageism had worse subjective health especially when they also had higher subjective age (Fig. 3). In addition, including the interaction terms rendered the main effect of continued growth significant (β = 0.08, *p* = 0.048), which was not significant in the step including the main effects only.

**Table 3:** Hierarchical regression results with subjective health as outcome variable

Variable	*B*	*SE B*	β	*p*	*R*^*2*^	*p*	Δ*R*^*2*^
*Step 1*					0.03	< 0.001	0.03*
Constant	5.19	0.31		< 0.001			
Age	-0.02	0.00	− 0.16	< 0.001			
*Step2*					0.04	0.003	0.02*
Constant	5.21	0.31		< 0.001			
Age	− 0.02	0.00	− 0.16	< 0.001			
Discrimination index	− 0.09	0.03	− 0.12	0.004			
*Step 3*					0.30	< 0.001	0.26*
Constant	4.76	0.28		< 0.001			
Age	− 0.01	0.00	− 0.10	0.011			
Discrimination index	− 0.04	0.03	− 0.06	0.147			
SPA Social loss	− 0.04	0.03	− 0.06	0.135			
SPA Continued growth	0.06	0.03	0.07	0.068			
SPA Physical decline	− 0.28	0.03	− 0.40	< 0.001			
Subjective age	− 0.10	0.03	− 0.14	0.001			
*Step 4*					0.33	< 0.001	0.04*
Constant	4.72	0.27		< 0.001			
Age	− 0.01	0.00	− 0.09	0.016			
Discrimination index	− 0.02	0.03	− 0.04	0.373			
SPA Social loss	− 0.05	0.03	− 0.07	0.097			
SPA Continued growth	0.06	0.03	0.08	0.048			
SPA Physical decline	− 0.29	0.03	− 0.41	< 0.001			
Subjective age	− 0.09	0.03	− 0.13	0.001			
Discr x Social loss	0.02	0.03	0.03	0.556			
Discr x Continued growth	0.04	0.03	0.04	0.301			
Discr x Physical decline	0.04	0.03	0.05	0.309			
Discr x Subjective age	− 0.14	0.03	− 0.19	< 0.001			

SPA Self-perceptions of aging**;** * *p* < .05

## Discussion

In light of the numerous reports of ageism during the Covid-crisis, our study provides empirical evidence from the perspective of older adults themselves. We focused on Luxembourgish older adults’ perception of having been treated unfairly due to their age during the first months of the pandemic. While levels of perceived ageism were relatively low for all domains assessed, around 20% of participants reported to have been treated somewhat unfairly due to their age in the media, health care and in their daily activities. This is comparable to data from Israel as reported by Cohn-Schwartz and Ayalon (2020). Besides, individual differences in perceived ageism mattered for participants’ subjective health and reported life satisfaction during the crisis: Those who reported to have experienced more ageism during the crisis also reported lower subjective health and life satisfaction after the onset of the crisis, even when controlled for reported life satisfaction before the onset of the crisis, which supports our first hypothesis. This is in line with previous studies linking perceived ageism to life satisfaction and self-rated health (Garstka et al. 2004; Vauclair et al. 2015). Given that both variables are important indicators of health and well-being (Whitley et al. 2016), this provides further evidence for the impact of perceived ageism on successful aging (Chang et al. 2020).

Albeit correlational, these findings support the numerous researchers and representatives who have warned about the negative effects of ageist narratives and actions during the pandemic (e.g. Ayalon et al. 2020, Meisner 2020; Wahl and Ehni 2020). While it is of course necessary and advisable for societies to protect its most vulnerable and in-need members, especially in times of heightened risk and crisis, simplifications in risk communication, ignoring individual differences within societal groups, and thereby fueling intergenerational conflict might have profound effects on individuals and also on societies as a whole in the long run.

Since negative age-related communication and actions have a larger impact on individuals when they have stronger self-relevance and are met with pre-existing negative perceptions about one’s own aging, we investigated the moderating role of self-perceptions of aging and subjective age. Supporting our hypotheses H2a and H2b, and in line with previous studies that show the relevance of subjective aging for development especially in the second half of life (e.g., Wurm et al. 2017), we found several main effects for the subjective aging variables on life satisfaction and subjective health. Those who perceived their own aging as accompanied by continued growth had better life satisfaction after the onset of the crisis and feeling younger than one’s actual age acted as a resource for better health. Contrary to other studies (Westerhof et al. 2014) it was however not related to life satisfaction. In line with assumptions regarding the domain-specificity of views on aging and the stereotype matching hypothesis (Levy and Leifheit-Limson 2009), self-perceptions of aging as social loss were only related to life satisfaction and self-perceptions of aging were only related to subjective health, i.e., the outcome domains that best matched their respective content. This supports our fourth hypothesis and again highlights the need for a differentiated assessment of views on aging when predicting diverse developmental outcomes (Kornadt et al. 2020).

In line with our hypotheses H3a and H3b, we found evidence that views on aging moderated the ageism—outcome relationship and thus can act as risk factors and resources in challenging situations. However, this was only true for subjective age and self-perceptions of aging as social loss, while, contrary to our expectations, self-perceptions of aging as continued growth and physical decline did not act as buffers or amplifiers for the relation between ageism and outcomes. Those who felt younger were less affected in their subjective health when they perceived ageism during the crisis. It seems that distancing oneself from ones’ age group and thus in many cases from the proclaimed risk population seems to be beneficial for health-related outcomes when there is a perceived threat to this group, at least in the short run. This is in line with previous experimental research (Weiss et al 2013). However, the question is whether there also might be negative consequences of such distancing on other indicators, which might compromise or facilitate the compliance with safety measures and risk perception (e.g., Bruine de Bruin and Bennett 2020). Paradoxically, excluding oneself from the risk group might actually lead to a heightened risk of actual infection; this possibility and the implications for older adults need to be addressed in future studies with different timelines.

Stronger endorsement of self-perceptions of aging as social loss also increased the negative relation of ageism and life satisfaction. Having a strong belief that one’s own aging is or will be accompanied by increases in loneliness, boredom and uselessness, as well as decreases in respect seems to increase older adults’ susceptibility to negative messages about their age group. This might be especially pronounced in the current pandemic, due to the focus on distance as a key factor in preventing the spread of the disease. Older adults with negative beliefs about aging regarding social loss might perceive the current situation and the negative societal atmosphere toward older adults as the starting point for inevitable, irreversible age-related decline. This in turn might lead to those perceptions becoming self-fulfilling prophecies (Levy 2009). Again, the long-term impact and the possibility of a rebound in life satisfaction after the preventive measures have been lifted need to be addressed in future studies.

### Limitations and implications for future research

While our study has several strengths, such as the large sample and the differential assessment of perceived ageism and subjective aging variables, some limitations have to be noted that at the same time point to opportunities for future research. First of all, our study is correlational in nature, thus, inferences about causality or directionality cannot be drawn. Even though the directional relationship between ageism and developmental outcomes in later life has been shown by previous longitudinal and experimental studies, older people with lower life satisfaction and subjective health might more readily perceive instances of ageism. Since the relationships might also differ according to the timeline applied (e.g., short-term, vs. weeks, vs. months), longitudinal studies with different time intervals are needed to pursue the long term consequences of ageism triggered by the Covid-crisis for the future (personal) development of older people.

Since our study was not prospective in nature, we did not have a value for life satisfaction at our disposal that was actually assessed before the onset of the pandemic, but had to rely on retrospective ratings, which might have been biased. However, we still consider them to be a good approximation, since they capture the discrepancies between the time before and after the onset of the pandemic as they were perceived by our participants at the time of the assessment. Relatedly, we did not have any other retrospective measure, for example for subjective health to control for, and also no other indicators of health problems, which might have impacted our findings. Besides, the perception of ageism is also dependent on factors we did not control for: For instance, participants with family members are at higher risk of perceiving ageism in the family compared to those without family; people who use health care more frequently may be more susceptible to ageism in this domain.^3^ These structural factors should be addressed in future research.

Even though we were careful to assess perceived ageism in a differentiated way and absolute numbers were similar to studies from other countries (Cohn-Schwartz & Ayalon, 2020), we asked people about “being treated unfairly” due to their age. Even though similarly worded questions have been used in other studies (e.g., Bratt et al 2018), this might put the focus on malevolent instances of ageism and not bring to mind benevolent ageism (such as the well-meant take-over of chores, regardless of people’s wishes), which might still have negative effects, perhaps more on an implicit and long-term level, for example by reducing autonomy and self-worth (Monahan et al. 2020). These effects also need closer inspection in future studies. Besides, even though our measure assessed perceived ageism in different domains, we decided to collapse them into one index for psychometric reasons and parsimony, at the expense of a differentiated view of ageism in different domains and on different levels (individual, societal). This is an important distinction and needs to be addressed in more detail in future studies (Ayalon and Tesch-Römer 2017, 2018).

Our sample consisted of community-dwelling, independently living, mostly middle-class older adults who reported to have good health and being rather satisfied with their lives. It will also be meaningful to address the questions of ageism in contexts where the pandemic and the protective measures taken might have an even stronger impact on older people’s health and well-being. This might be true for older people living in unfavorable (e.g., in poverty, poor health, violent contexts) and also in institutionalized contexts. Since much press coverage was also centered at older adults in homes or hospitals, they might bear the double burden of increased risks from the disease and ageism even stronger.

### Conclusion

To conclude, our study provides one of the first empirical investigations of perceived ageism and views on aging during the Covid-crisis and its relationship to the health and well-being of older people. The perception of ageism might affect this demographic group profoundly, especially in times of uncertainty and hardship. Propositions to mitigate ageism and its consequences in the crisis have been made (e.g., Wahl and Ehni 2020) and need to find application in management of risk communication, intergenerational relationships and support as well as public discourse. For instance, the communication enhancement model of aging proposed by (Ryan and colleagues 1995) might be used to develop non-discriminatory risk communication and coping strategies: Communicating in a non-discriminatory manner the necessity of preventive measures, and at the same time recognizing the diversity of older people, their resources, experiences, competences and needs, as well as their role as active and valuable members of society. This approach should result in a more productive and benevolent societal discourse and more favorable outcomes for individuals and (aging) societies alike, especially but not only in times of crisis.

## Data Availability

All data and materials can be obtained from the authors upon request.

## Footnotes

1 We also assessed perceived ageism in the work context. However, due to the fact that 90% of our participants were already retired, this domain was not relevant for most and thus, *n* = 372 participants (61%) did not report on this item. Therefore, we excluded this item from the index. Results did not differ for both versions of the index.

2 Results for both regression analyses did not change when including gender, education, living situation (alone vs. with others) and survey mode as additional covariates.

3 We thank an anonymous reviewer for pointing out this important point.

## Code availability

The SPSS syntax used for all analyses can be obtained from the first author upon request.
